# Melanocortin-4 Receptor PLC Activation Is Modulated by an Interaction with the Monocarboxylate Transporter 8

**DOI:** 10.3390/ijms25147565

**Published:** 2024-07-10

**Authors:** Larissa Anthofer, Philipp Gmach, Zeynep Cansu Uretmen Kagiali, Gunnar Kleinau, Jonas Rotter, Robert Opitz, Patrick Scheerer, Annette G. Beck-Sickinger, Philipp Wolf, Heike Biebermann, Ingo Bechmann, Peter Kühnen, Heiko Krude, Sarah Paisdzior

**Affiliations:** 1 Institute of Experimental Pediatric Endocrinology, Charité—Universitätsmedizin Berlin, Freie Universität Berlin, Humboldt-Universität zu Berlin, D-10117 Berlin, Germany; 2 Institute of Anatomy, Leipzig University, D-04103 Leipzig, Germany; 3 Group Structural Biology of Cellular Signaling, Institute of Medical Physics and Biophysics, Charité—Universitätsmedizin Berlin, Freie Universität Berlin, Humboldt-Universität zu Berlin, D-10117 Berlin, Germany; 4 Faculty of Life Sciences, Institute of Biochemistry, Leipzig University, D-04103 Leipzig, Germany; 5 Department for Pediatric Endocrinology and Diabetology, Charité—Universitätsmedizin Berlin, Freie Universität Berlin, Humboldt-Universität zu Berlin, D-10117 Berlin, Germany

**Keywords:** protein–protein interaction, heterodimerization, MC4R, MCT8, fluorophore-labeled ligand, BRET

## Abstract

The melanocortin-4 receptor (MC4R) is a key player in the hypothalamic leptin–melanocortin pathway that regulates satiety and hunger. MC4R belongs to the G protein-coupled receptors (GPCRs), which are known to form heterodimers with other membrane proteins, potentially modulating receptor function or characteristics. Like MC4R, thyroid hormones (TH) are also essential for energy homeostasis control. TH transport across membranes is facilitated by the monocarboxylate transporter 8 (MCT8), which is also known to form heterodimers with GPCRs. Based on the finding in single-cell RNA-sequencing data that both proteins are simultaneously expressed in hypothalamic neurons, we investigated a putative interplay between MC4R and MCT8. We developed a novel staining protocol utilizing a fluorophore-labeled MC4R ligand and demonstrated a co-localization of MC4R and MCT8 in human brain tissue. Using in vitro assays such as BRET, IP1, and cAMP determination, we found that MCT8 modulates MC4R-mediated phospholipase C activation but not cAMP formation via a direct interaction, an effect that does not require a functional MCT8 as it was not altered by a specific MCT8 inhibitor. This suggests an extended functional spectrum of MCT8 as a GPCR signaling modulator and argues for the investigation of further GPCR-protein interactions with hitherto underrepresented physiological functions.

## 1. Introduction

The melanocortin-4 receptor (MC4R) is a key component of the leptin–melanocortin pathway, which regulates energy homeostasis in the hypothalamus [1]. Mainly expressed in the paraventricular nucleus (PVN) of the hypothalamus, this receptor plays an important role in the regulation of hunger and satiety [2].

Endogenous ligands of MC4R are proopiomelanocortin (POMC)-derived peptides, such as the α-, β- or γ-Melanocyte-stimulating hormone (MSH) [3]. After the extracellular binding of an agonistic ligand, MC4R activates the G_s_/adenylyl cyclase pathway [3], the G_q/11_/phospholipase C (PLC) pathway [4,5,6], and G_i/0_ [7]. While Gs-deficient mice become obese without hyperphagia and decreased energy expenditure, Gq/11-deficient mice display increased food intake, resulting in obesity [5,8]. In contrast, activation of G_i/0_ mediates orexigenic effects [7].

MC4R has been shown to form homodimers [9,10,11] as well as heterodimers with other GPCRs such as the G protein-coupled receptor 7 and the serotonin 1B receptor [12]. Furthermore, MC4R interacts with the melanocortin-2 receptor accessory protein 1 (MRAP1) and MRAP2 [13], which are factors modulating the ligand sensitivity and cAMP signaling capacity of MC4R [14]. Generally, oligomerization can have a significant impact on GPCR properties such as its trafficking to the cell surface, ligand binding, G-protein coupling, and agonist-induced internalization [15].

A further membrane-spanning protein metabolically connected with the leptin–melanocortin pathway is the monocarboxylate transporter 8 (MCT8). Encoded by the *SLC16A2* gene on the human Xq13.2 locus [16], MCT8 is the most specific transporter of thyroid hormones (TH) [17]. The main substrate of MCT8, triiodothyronine (T3), has an immense variety of functions and affects almost every physiological process in the body [18]. It probably also stimulates food intake in humans by, including but not limited to, reducing POMC expression [19], and is therefore directly involved in the leptin–melanocortin pathway and central actions in the hypothalamus [20].

MCT8 is a member of the Major Facilitator Superfamily (MFS), the largest family of membrane transport proteins which facilitate the transport of solutes across cell membranes [21]. Structurally, MCT8 is characterized by 12 transmembrane helices which are connected by extra- and intracellular loops [22]. This transporter is highly abundant in endothelial cells of brain barriers but can also be found in neurons and astrocytes of the paraventricular and arcuate nuclei of the human hypothalamus [23,24].

It has been demonstrated that MCT8 is able to form homodimers [25,26], which is crucial for its function as a substrate transporter [27]. In addition, heterodimerization with the thyroid-stimulating hormone receptor (TSHR), a class A GPCR, leads to a bias in the TSHR signaling profile, namely a reduction specifically in G_q/11_-signaling, but not in Gs-mediated cAMP accumulation [21]. Of note, equivalent to MCT8 and TSHR, several members of the MFS have been shown to interact with GPCRs, e.g., Glucose Transporter 4 and the dopamine transporter [28,29].

Based on the observations of GPCR–transporter interactions, including the known MCT8–TSHR interaction [21], the capacity of MC4R to oligomerize [9,10,11], and the fact that T3 as a substrate of MCT8 is also metabolically connected to the leptin–melanocortin pathway [30,31], we hypothesized a potential direct interaction between both proteins in heteromeric complexes. Therefore, we analyzed MC4R and MCT8 co-expression and investigated whether MCT8 modulates the signaling capacity of MC4R.

We found a co-localization of both proteins in primary human hypothalamic brain tissue, demonstrated a specific interaction of MC4R and MCT8 in vitro, and discovered that MCT8 does not modify MC4R-Gs-dependent cAMP formation but modulates MC4R-dependent PLC activation in a concentration-dependent manner. This points to a potential allosteric impact of MCT8 on MC4R, leading to more specific instead of promiscuous intracellular signaling induction.

## 2. Results

### 2.1. Evidence of Co-Expression and Co-Localization of MC4R and MCT8 in Neurons of the Hypothalamic Paraventricular Nucleus 

Using a publicly available data set of single-cell RNA-sequencing (scRNAseq), we found co-expression of the two genes, *MC4R* and *SLC16A2*, the MCT8-encoding gene, in neurons of dissected human hypothalamus (dataset from [32], Supplementary Figure S1). Nuclei of several hypothalamic regions were dissected and sequenced, including the PVN. While most sequenced cells had neuronal identity, sorting for *SLC16A2*-expressing cells revealed MCT8 expression in various cell types, including neurons, epithelial cells, and oligodendrocytes. Additional sorting for *MC4R*-expressing cells, which were of neuronal identity only, showed expression in fewer cells. Within this *MC4R*-expressing subpopulation of neurons, we sorted for *SLC16A2* expression to single out *MC4R* and *SLC16A2* co-expressing neurons. We found that around 40% of the *MC4R*-expressing cells co-express *SLC16A2*. As the dataset includes not only the PVN but also part of the preoptic and supraoptic regions, we aimed to investigate the co-expression of MC4R and MCT8 specifically in human PVN tissue.

We thus set out to assess for a co-localization of both proteins using human brain tissue, namely of the PVN, obtained from deceased body donors (Institute for Anatomy, University of Leipzig) and applied a novel staining technique to visualize MC4R and MCT8 localization. Due to the lack of validated antibodies for MC4R, we employed an innovative staining technique based on a fluorophore-labeled ligand of MC4R, Carboxytetramethylrhodamine (TAMRA)-[Nle^4^, DPhe^7^]-(NDP)-α-MSH, in addition to an antibody-staining of MCT8 (for the specificities of the fluorophore-labeled MC4R-ligand and the anti-MCT8 antibody, see our previously published data [33,34]). 

**Figure 1:**
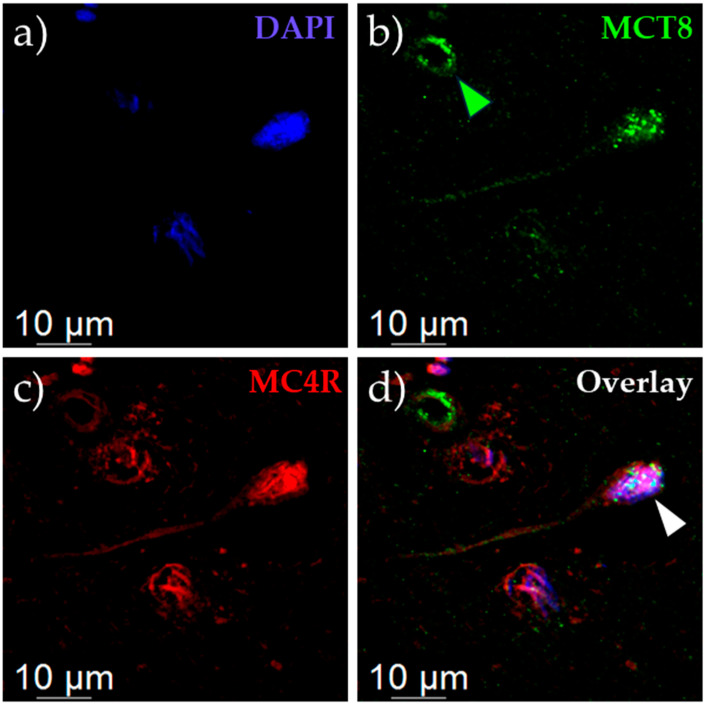
**Staining of MCT8 and MC4R in tissue of the human paraventricular hypothalamus.** Tissue was stained with anti-MCT8 ((**b**), green) and TAMRA-NDP-α-MSH ((**c**), red) to enable MC4R staining. Nuclei were counterstained with DAPI ((**a**), blue). In addition to extensive MCT8 staining in endothelial cells ((**b**), top left, indicated by green arrow), MCT8 and MC4R co-staining was found in neurons ((**d**), purple soma indicated by white arrow).

Generally, there was less MC4R staining than MCT8, with MCT8 staining, as expected [25], abundantly found in endothelial cells of the blood vessels (Figure 1b, green), while MC4R staining was absent in these structures. In neurons, a co-localization of MCT8 and MC4R was present (Figure 1c, red), mainly in the soma and axons as identified by the long structure of axons and conic-shaped soma (Figure 1d, co-localization in purple, indicated by white arrow). In line with other reports that demonstrate no evidence for MC4R expression within the PVN outside of neurons [35], we did not observe a co-localization of both proteins in cells of obvious non-neuronal identity, e.g., endothelial cells of blood vessels.

### 2.2. MCT8 Interacts with MC4R In Vitro 

**Figure 2:**
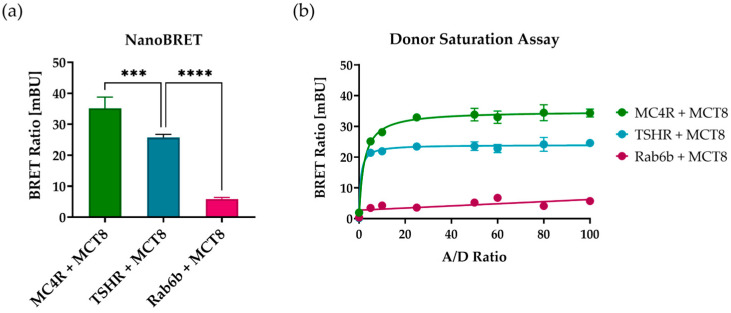
**Investigation of MC4R–MCT8 heterodimer formation.** An interaction between MC4R and MCT8 was analyzed with the NanoBRET™ assay using C-terminally tagged constructs. (**a**) HEK293 cells were co-transfected with the BRET partners MCT8-HaloTag (HT) and NanoLuciferase (NL)-tagged MC4R/TSHR/Rab6b. The close proximity of the BRET partners results in energy transfer manifesting in a high BRET ratio. While the negative control pair Rab6b–MCT8 demonstrated a low BRET ratio, the pair under investigation (MC4R–MCT8) showed a higher BRET ratio than the positive control TSHR-MCT8. Values represent mean ± SEM from four independent experiments with three technical replicates. Individual values from technical replicates were pooled and the mean was used for analysis. A one-way ANOVA was performed for statistical analysis and the mean of the positive control TSHR–MCT8 was compared to the mean of all other BRET pairs. Statistical significance was defined as *** *p* < 0.001 and **** *p* < 0.0001. (**b**) A Donor Saturation Assay was performed to investigate the specificity of the MC4R–MCT8 interaction. HEK293 cells were transfected with an increasing amount of donor (MC4R-NL, Rab6b-NL, TSHR-NL) while keeping the amount of acceptor (MCT8-HT) constant. The positive control (TSHR–MCT8) and MC4R–MCT8 demonstrated a specific interaction, while the negative control (Rab6b–MCT8) resulted in a linear increase in the BRET ratio, demonstrating a non-specific interaction. Values represent mean ± SEM from two independent experiments.

To investigate an interaction between MC4R and MCT8 in vitro, we determined heterodimerization of MCT8 and MC4R with the NanoBRET™ assay in HEK293 cells (Figure 2a). The small G-protein Ras-related protein Rab6b was selected as a suitable negative control since it is, among other tissues, ubiquitously expressed in the human brain, where its expression is also found in neurons [36]. It interacts with a variety of effectors related to the G-protein signaling pathway [37]. As a positive control, TSHR, a thyroid-located GPCR that has been demonstrated to interact with MCT8, was used.

A strong BRET signal was observed for the combination of NanoLuc^®^ (NL)-tagged MC4R and HaloTag^®^ (HT)-tagged MCT8, resulting in a six-fold increase in BRET ratio in comparison to the negative control pair Rab6b-NL and MCT8-HT. The positive control with donor-TSHR and acceptor-MCT8 demonstrated a BRET ratio close to the ratio of MC4R–MCT8 complexes. A Donor Saturation Assay was performed for all three combinations, which verified the specificity of the interaction between MC4R–MCT8 and TSHR–MCT8 (Figure 2b).

### 2.3. MCT8 Co-Expression and Interaction with MC4R Does Not Influence Gs-Signaling 

**Figure 3:**
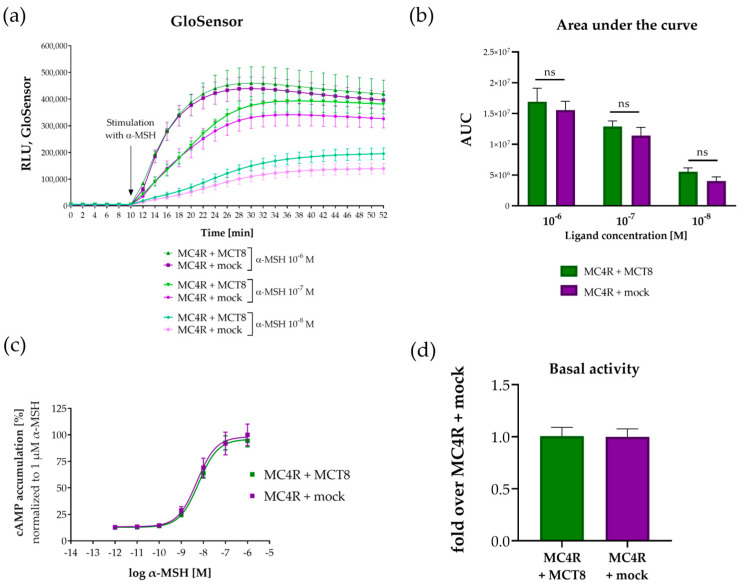
**MC4R-mediated cAMP formation in the presence and absence of MCT8.** (**a**) Time-dependent changes in GloSensor™ cAMP luminescence after stimulation with α-MSH were determined. HEK293 cells were co-transfected with MC4R + mock or MC4R + MCT8 in addition to luciferase-containing F22 reporter plasmid, stimulated with α-MSH in concentrations ranging from 10^−6^ M to 10^−8^ M (time point of stimulation indicated by black arrow), and cAMP formation was measured. No significant differences in cAMP concentrations were observed in the absence or presence of MCT8, shown by the calculated area under the curve (**b**). Values represent mean ± SEM from four independent experiments. Each experiment contained six biological replicates, whose individual values were pooled for analysis. Statistical analysis was performed using an ordinary two-way ANOVA with Sidak’s multiple comparison test with statistical significance set at *p* < 0.05, nonsignificant marked as ns. (**c**) Concentration–response curve of MC4R-mediated cAMP accumulation after stimulation with α-MSH in concentrations ranging from 10^−12^ M to 10^−6^ M. HEK293 cells were co-transfected with MC4R + mock or MC4R + MCT8 and cAMP accumulation was measured using the AlphaScreen^®^ assay. No differences were detected in MC4R-dependent cAMP formation in the presence of MCT8 compared to its absence, neither when stimulated nor in basal conditions (**d**). Concentration–response curves were analyzed by fitting a non-linear regression model for sigmoidal response. Values represent individual measurement values ± SD from four independent experiments with three biological replicates.

After obtaining evidence for a MC4R–MCT8 dimer formation, we set out to determine whether this dimerization has a functional influence on the signaling capacity of MC4R. As the most significant pathway, we first tested the G_s_-dependent cAMP formation of MC4R in the presence or absence of MCT8 in transfected HEK293 cells (Figure 3). The signaling properties were evaluated using the GloSensor™ assay to determine acute cAMP production resulting from receptor stimulation with α-MSH (Figure 3a). After measurement of the basal activity level, addition of α-MSH resulted in concentration-dependent cAMP accumulation. The area under the curve (AUC) for cells transfected with MC4R and MCT8 compared to cells transfected with MC4R and mock was determined. No significant differences in the AUC were detected in the presence of MCT8 compared to its absence (Figure 3b). To exclude that the presence of MCT8 leads to a partial loss of function of MC4R, we performed a concentration–response curve for MC4R-dependent cAMP accumulation in the presence and absence of MCT8. Here, we detected no significant differences, neither in potency (EC_50_(MC4R + mock) = 5.29 ± 1.33 nM vs. EC_50_(MC4R + MCT8) = 6.11 ± 0.25 nM) nor in efficacy (E_max_(MC4R + mock) = 7.73 ± 0.07 vs. E_max_(MC4R + MCT8) = 7.29 ± 0.43-fold over MC4R + mock) (Figure 3c). We found no differences in MC4R basal activity in the presence of MCT8 compared to its absence (Figure 3d). Therefore, we conclude that the presence of MCT8 has no effect on the Gs-signaling properties of MC4R.

### 2.4. MCT8 Modulates MC4R-Mediated PLC Activation in a Concentration-Dependent Manner 

**Figure 4:**
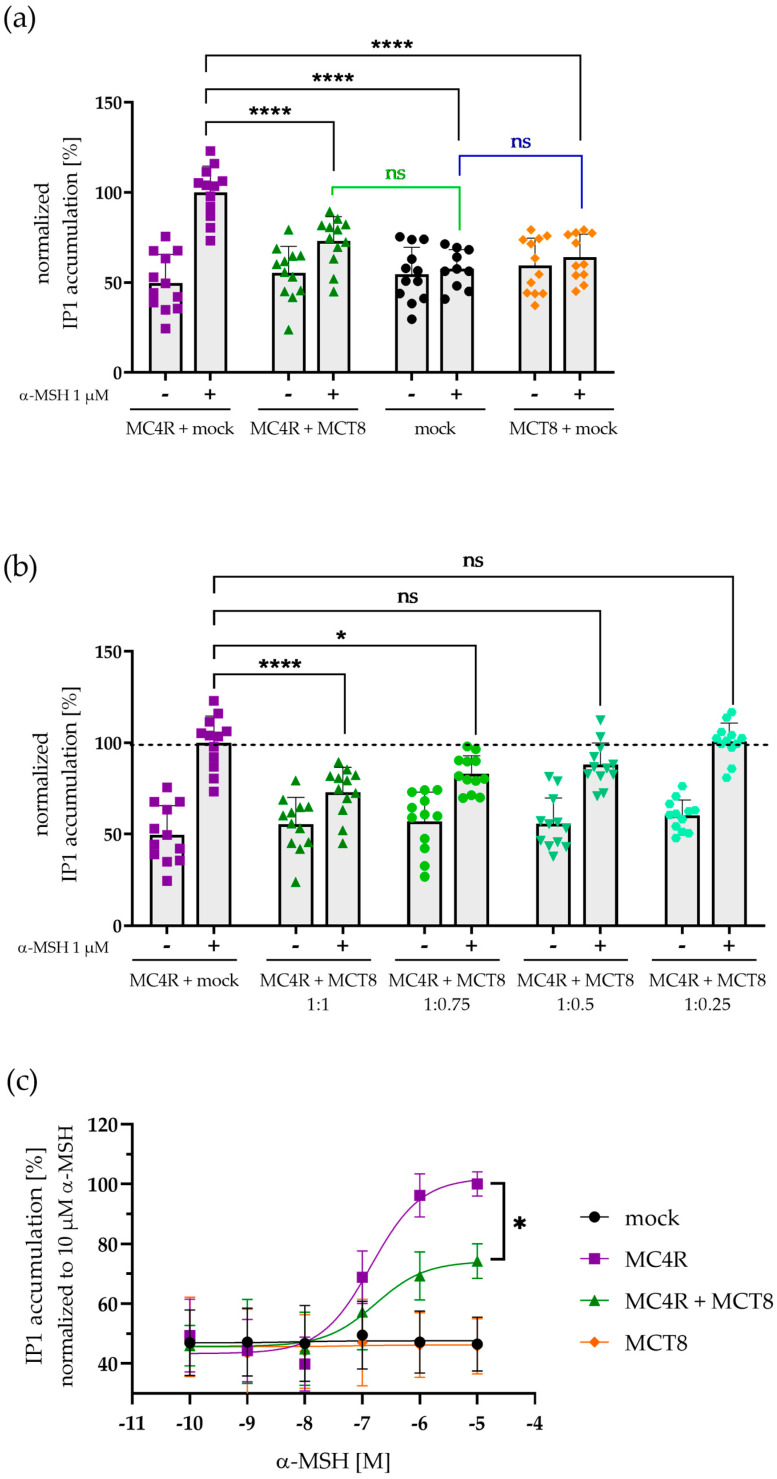
**MC4R-mediated IP1 accumulation after stimulation with α-MSH is dependent on the presence and concentration of MCT8.** (**a**) HEK293 cells were co-transfected with MC4R + mock or MC4R + MCT8 and IP1 accumulation was measured in basal conditions and after stimulation with 1 µM α-MSH. IP1 concentrations were calculated using a standard curve and normalized to MC4R + mock. There were no significant differences in IP1 formation in basal conditions. When stimulated with 1 µM α-MSH, significant differences in IP1 concentrations were detected in the presence of MCT8 (black asterisks). Mock- and MCT8 + mock-transfected samples (blue line) along with MC4R + MCT8- and mock-transfected samples (green line) demonstrated no significant differences in IP1 accumulation. (**b**) HEK293 cells were co-transfected with MC4R + mock or MC4R + MCT8 in different ratios (indicated on the x-axis) and stimulated with 1 µM α-MSH. With decreasing concentrations of MCT8, significant differences in IP1 accumulation were no longer present, and IP1 concentrations approached MC4R + mock-transfected levels again. Values represent individual measurement values ± SD from four independent experiments with three technical replicates. After testing for normal distribution of the data, a one-way ANOVA was performed for statistical analysis, and Holm–Sidak’s multiple comparison test was performed by comparing the values of MC4R + mock to other values. Statistical significance was defined as * *p* < 0.05 and **** *p* < 0.0001. (**c**) HEK293 cells were co-transfected with MC4R + mock or MC4R + MCT8 in a 1:1 ratio and IP1 accumulation was measured after stimulation with α-MSH in concentrations ranging from 10^−5^ M to 10^−10^ M. IP1 concentrations were calculated analogously to above. The presence of MCT8 resulted in a significant decrease in the efficacy of MC4R-mediated PLC activation. Values represent individual measurement values ± SD from four independent experiments with three technical replicates. Statistical analysis was performed using an unpaired one-way ANOVA with the Kruskal–Wallis test to compare the E_max_ of MC4R + mock and MC4R + MCT8. Statistical significance was defined as * *p* < 0.05, nonsignificant marked as ns.

Since we and others described a relevant G_q/11_-signaling function of the MC4R [4,5,6,8], we further investigated the PLC activation capacity of MC4R in the presence or absence of MCT8. HEK293 cells were transfected with either an empty mock plasmid, MC4R and mock, or combinations of MC4R and MCT8. IP-One assays were conducted to measure inositol monophosphate (IP1) accumulation in basal conditions and after stimulation with 1 µM α-MSH (Figure 4). IP1 concentrations were calculated using the homogenous time-resolved fluorescence (HTRF^®^) ratio obtained from acceptor emission measurement using a standard curve.

Without stimulation (basal condition), IP1 formation remained constant and independent of the presence of MCT8 (Figure 4a). We therefore concluded that MCT8 has no influence on the basal level of MC4R-mediated PLC activation.

Interestingly, when stimulated with 1 µM α-MSH, MC4R-mediated IP1 formation was significantly reduced by 27% when MCT8 was present compared to the absence of MCT8 (Figure 4a). This effect was shown to be dependent on the concentration of MCT8, as IP1 formation increased again once MCT8 was present in decreasing amounts (Figure 4b).

Concentration–response experiments with α-MSH in the presence and absence of MCT8 confirmed a reduced PLC activation in the presence of MCT8 (Figure 4c). While the EC_50_ remained similar (EC_50_(MC4R + mock) = 145.19 ± 33.4 nM vs. EC_50_(MC4R + MCT8) = 192.79 ± 89.96 nM), the E_max_ was significantly reduced by 26% (100% ± 3.90% in the absence of MCT8 vs. 74.19% ± 5.55% in the presence of MCT8, *p* = 0.0158).

To investigate whether this effect is dependent on a functional MCT8, we determined MC4R-elicited IP1 formation in the presence of MCT8 and 10 µM of the MCT8-specific inhibitor silychristin (SC, [38]) (Figure S2a). The reducing effect on PLC activation seen in the other assays persisted even in the presence of SC, with no differences in IP1 accumulation detected in basal or stimulated conditions.

As a control, IP1 formation was also assessed after transfection with MC4R in the presence or absence of the cannabinoid receptor 1 (CB1R), a GPCR that is suspected not to interact with MC4R [39]. HTRF^®^ ratios and IP1 formation remained constant in all stimulated and basal conditions and were independent of the presence of CB1R (Figure S2b). Hence, the decrease in PLC activation by the presence of MCT8 seems specific to the interaction of MC4R and this transporter.

### 2.5. Interaction between MC4R and MCT8 Does Not Modulate Their Cell Surface Expression 

**Figure 5:**
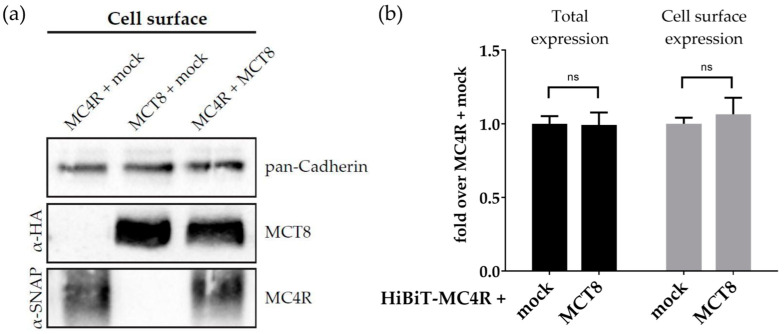
**Analysis of MC4R and MCT8 cell surface expression with biotinylation and HiBiT assays.** (**a**) HEK293 cells were transfected with SNAP–MC4R + mock, HA–MCT8 + mock, or co-transfected with SNAP–MC4R + HA–MCT8. Western blotting of biotinylated cell surface proteins demonstrated no change in the cell surface expression of MC4R or MCT8 in the presence or absence of one another. Pan-Cadherin, a plasma membrane marker protein, was used as a loading control. (**b**) HEK293 cells were co-transfected with HiBiT–MC4R + mock or HiBiT–MC4R + MCT8. The cell surface and total expression levels of HiBiT-MC4R did not show a significant change in the presence of MCT8 compared to its absence. Values represent mean ± SEM from three independent experiments. A two-way ANOVA was performed for statistical analysis with Dunn’s multiple comparison test. Nonsignificant marked as ns.

To understand the mechanism behind the observed reduction in PLC activation, we investigated whether this modulated signaling is due to the altered expression of either protein on the cell surface. HEK293 cells were transfected with SNAP-tagged MC4R (SNAP–MC4R) and mock, Hemagglutinin (HA)-tagged MCT8 (HA-MCT8) and mock, or SNAP–MC4R and HA–MCT8. Biotinylated cell surface proteins were isolated and subjected to Western blot analysis. Compared to the individual expressions of MC4R and MCT8, the co-expression of MC4R and MCT8 did not alter the protein levels of one another on the cell surface (Figure 5a). Western blot analysis of total lysates of the transfected cells revealed that the total protein levels of MC4R and MCT8 were also similar in the presence or absence of one another (Figure S3).

These findings were supported by the High Binary Technology (HiBiT) assay (Figure 5b), a technique used to investigate total and cell surface expression via a split luciferase. Compared to the absence of MCT8, there were no significant differences detected for HiBiT–MC4R expression in the presence of MCT8, neither in total cell lysates nor on the cell surface.

## 3. Discussion

The interaction between GPCRs and other proteins can modulate the trafficking, signaling, or ligand-binding properties of the receptor, therefore adding another level of functional control [40]. While some examples of interactions between GPCRs and proteins of other families, e.g., ligand- or voltage-gated channels, receptor activity modulating proteins (RAMPs), or accessory proteins are known [15,41], there are fewer examples of GPCRs interacting with substrate transporters. Such interactions have been demonstrated for instance for the β2-adrenergic receptor (ADRB2) and the glucose transporter 4 [28], or for the trace-amine-associated receptor 1 and dopamine transporter [29]. Our working group has also shown an interaction of the TSHR, a class A GPCR, and the TH transporter MCT8 at a single-molecule level [21]. An interaction of GPCRs such as MC4R and substrate transporters such as MCT8 could enable cross-talk between different physiological pathways in the human body [42], in this case the leptin–melanocortin pathway and the hypothalamus–pituitary–thyroid axis, thus opening the possibility for fine-tuning of two pathways at once.

In this current study, we described the interaction of the GPCR MC4R and the TH transporter MCT8. We showed a co-staining of MC4R and MCT8 in human brain tissue (Figure 1), demonstrating a concurrent protein expression in human PVN neurons using a combination of fluorophore-labeled ligands and antibodies. To the best of our knowledge, the co-staining of human brain tissue by employing a fluorophore-labeled ligand and antibodies has never been demonstrated before. Hence, this approach provides a solid foundation for novel advanced staining procedures. In combination with our analysis of scRNAseq data of the human hypothalamus (Figure S1), we can conclude that co-expression of MC4R and MCT8 exists in neurons of the PVN, however, they are not exclusively expressed together. In an in vitro system with overexpressing HEK293 cells, NanoBRET™ assays show that MC4R and MCT8 are indeed interacting (Figure 2). Since BRET methods count as a superior method for the investigation of protein interactions due to the necessity of spatial proximity of the proteins for BRET to occur and the possibility to be performed in live cells [41], the results of our assays demonstrate a specific interaction between MC4R and MCT8.

Further investigation of a functional influence of the MCT8 interaction with MC4R revealed a significantly decreased α-MSH-induced PLC activation for MC4R manifesting as reduced IP1 accumulation in co-transfected HEK cells, while the basal IP1 level was not affected (Figure 4). This effect is a sole efficacy-reducing effect, which does not involve a shift in potency. Noteworthy, PLC activation levels increased again when MCT8 was diluted, showing that this effect on MC4R signaling is specific to the presence and decreasing concentration of MCT8.

Modulation of GPCR signaling capacity after heteromerization has been observed numerous times, although it can lead to either a rise or a reduction in G-protein signaling [15]. For instance, heterodimerization of another melanocortin receptor, MC3R, with the growth hormone secretagogue receptor (GHSR) results in enhanced Gs-signaling of MC3R while simultaneously reducing the G_q/11_-signaling of GHSR [43]. Heterodimerization of type I angiotensin II receptor with the bradykinin receptor increases angiotensin receptor-mediated G_q/11_- and G_i/0_-signaling [44]. Notably, our group already reported an interaction of MCT8 with the TSHR that resulted in a specific reduction of TSHR-mediated G_q/11_ signaling when co-expressed [21]. TSHR, like MC4R, is a promiscuous GPCR that shows responsiveness for several G-protein subtypes [45,46].

However, apart from excluding effects on cell surface or total expression, the underlying mechanism behind the influence on GPCR signaling capacity by the interaction with the transporter protein has not been determined so far. Putative mechanisms for the functional impact of the MCT8 on the MC4R which can be concluded from our data are discussed below.

*Homodimer formation:* MC4R and MCT8 are both known to form homodimers [9,25,26,47] or even oligomers in the case of MCT8 [25,26], that shape the functionality of the proteins. For the TSHR, homodimers are specifically crucial for G_q/11_ signaling [48], which is modulated by the interaction with MCT8 [21]. Conversely, for MC4R, potential dimer separation by interaction partners such as MRAP2 enhances the Gs-signaling capacity, presumably by stoichiometrically increasing the availability of MC4R for G-protein binding [49]. Recently, we showed that dimer separation has a comparable effect on G_q/11_-signaling [39]. Although our BRET assays (Figure 2) do not provide any information about the number of proteins involved in the interaction, the fact that PLC activation is inhibited rather than enhanced in this current study suggests that the interaction with MCT8 favors MC4R homodimers rather than monomers.

*Internalization and trafficking:* As seen in numerous GPCRs, interactions with other proteins can influence receptor internalization or membrane trafficking, mostly triggered by β-arrestins [50]. For instance, dimerization of the ADRB2 with the δ opioid receptor leads to its ligand-mediated endocytosis, while dimerization of the ADRB2 with the κ opioid receptors does not promote receptor internalization [51]. Additionally, heterodimerization between ADRB1 and ADRB2 inhibits the ligand-induced internalization of ADRB2 [46]. To potentially uncover the underlying mechanism of the observed reduction in PLC activation, we analyzed MC4R cell surface and total expression in HEK293 cells co-expressing MCT8 (Figure 5). Even though cellular expression and receptor trafficking are distinct processes, receptors with altered trafficking properties would disappear in the surface fraction, thus indicating receptor internalization. As we found no influence of MCT8 on MC4R total or cell surface expression, we conclude that MCT8 does not modulate unstimulated MC4R expression or membrane trafficking. This is supported by the observed unaffected Gs-signaling (Figure 3), as reduced expression would most likely influence all signaling pathways.

*Structural impact:* A further option could be that the reduction of IP1 formation by decreased G_q/11_ activation due to co-expressed MCT8, as observed here, results from conformational changes of the receptor caused by the interplay with MCT8. Such suspected, yet unknown interactions could inhibit specific receptor regions important in G_q/11_-coupling or alter dynamic and kinetic properties that enable decreased association of G_q/11_ proteins but are not essential for Gs-binding and -activation. Unfortunately, from a structural perspective, information regarding putative MCT8 homodimers, MC4R homodimers, or even putative MCT8–MC4R heteromers is completely absent so far [15,27,52]. Moreover, while the arrangement and binding mode of Gs at MC4R have been elucidated by the determination of MC4R-ligand-G_s_ complexes via cryo-electron microscopy, the binding mode of G_q/11_ is not solved in molecular detail yet [53].

However, the observed reduction of PLC activation at MC4R by MCT8 co-expression could be of physiological relevance for the specific modulation of MC4R function in the PVN [54]. Our data suggest that the amount of MCT8 expressed in the PVN could have an impact on weight regulation if the interaction with MC4R observed in vitro also occurs in vivo, e.g., with an increase in appetite due to higher MCT8 expression, resulting in increased T3 transport and therefore enhanced energy metabolism. As the two proteins are not exclusively expressed together in the PVN (Figure S1), we suspect MCT8 to be an additional co-regulator of MC4R signaling, adding another level of energy metabolism control in the hypothalamus.

So far, MCT8 is known to function solely as a transporter, as MCT8 is one of the best-characterized, major transporters for T3 throughout different species [17,55]. The diminishing effect of MCT8 on MC4R-mediated PLC activation persisted even in the presence of the specific MCT8 inhibitor SC (Figure S2a), indicating that active TH transport by MCT8 is not a prerequisite for the reducing effect on PLC activation and that the protein–protein interaction itself is sufficient to reduce G_q/11_-coupling to MC4R. Whether the heteromer formation affects MCT8 function remains to be elucidated and needs to be the subject of a follow-up study. For now, our finding of an interaction between MC4R and MCT8 and the subsequent impact on PLC activation thus suggests an additional physiological role of MCT8 by interacting with GPCRs, which might be independent of TH transport. Moreover, the view of further MCT8 functions besides T3 transport would be compatible with observations made in patients with MCT8 loss-of-function mutations who are affected by Allan–Herndon–Dudley Syndrome (AHDS), a severe developmental disease that is argued to be caused by the lack of T3 transport due to the disturbed transport function of MCT8 itself [56]. However, AHDS patients develop severe cachexia during their childhood [57], which, besides other mechanisms such as impaired T3 transport, could be the result of a disturbed interaction of the mutant MCT8 protein with MC4R.

Very recently, several studies investigated the effects of fasting on thyrotropin-releasing hormone (TRH)-expressing neurons in the PVN [30,31], which overlap with MC4R-expressing cells [58]. Interestingly, fasting increases TH concentrations by central and peripheral mechanisms [30], including the activation of Agouti-related peptide/Neuropeptide Y (AgRP/NPY) neurons, which counteract MC4R signaling [59]. Although there was a clear connection between the regulation of metabolism by TH in the PVN and the leptin–melanocortin pathway, details such as the identity of the mediating TH receptor remain elusive [31]. Combining these studies with our findings, it would be of interest to investigate the role of MCT8 during fasting or obesity. Here, future studies could incorporate, e.g., mouse models with an MC4R-dependent MCT8-knock out.

Taken together, we demonstrated a specific interaction between MCT8 and MC4R through advanced staining and BRET methods in vitro and in human brain tissues. The presence of MCT8 selectively reduces MC4R-dependent PLC activation, thereby acting as a modulator of MC4R action. Although the underlying mechanism of this interaction requires further elucidation, the findings could have a physiological impact and point to an additional function of MCT8 as a GPCR modulator.

## 4. Materials and Methods

### 4.1. Analysis of Single-Cell RNA-Sequencing Data 

We analyzed publicly available scRNAseq data using the CELLxGENE Discover tool [60] (accessed on 30 March 2023). The analyzed dataset was part of the Human Brain Cell Atlas v1.0 [32]. We chose a dissection that included the paraventricular nucleus, as well as other nuclei such as the preoptic region, the medial preoptic nucleus, and the supraoptic region. To mark *SLC16A2*- or *MC4R*-expressing cells, we selected the appropriate cells within this dataset with a lower mean expression cut-off set to 1 and displayed this subset. Co-expressing neurons were identified by selecting *MC4R*-expressing cells first, then marking *SLC16A2*-expressing cells within this selected subset (again with a lower mean expression cut-off at 1). As the tool allows for cell counting within the subset, we were able to calculate the amount of co-expressing cells.

### 4.2. Immunofluorescence and Fluorophore-Labeled Ligand Staining 

For the detection of MC4R, we employed TAMRA-NDP-α-MSH in addition to an antibody-based staining of MCT8. Staining with fluorophore-labeled ligands provides some advantages, as the staining procedure is more rapid compared to conventional staining with antibodies (20 min with fluorophore-labeled ligands compared to several hours using primary and secondary antibodies).

NDP-α-MSH was synthesized by solid phase peptide synthesis as described previously [61,62]. TAMRA was coupled to the N-terminus of the peptide on the resin. The fluorescent peptide was cleaved from the resin with trifluoroacetic acid and purified by high-pressure liquid chromatography. Identity was confirmed by electrospray ionization mass spectrometry.

All body donors or their relatives provided a written declaration of consent to explore the cadavers for research purposes. The utilization of human tissues for scientific purposes is authorized by the Ethics Committee of the University of Leipzig (129-21 ek). Human brain tissue of the paraventricular nucleus was obtained from a deceased body donor (male individual of 77 years, post-mortem interval: <24 h) and embedded in paraffin. Serial sections with 9 µM thickness were cut on a microtome (Leica, Wetzlar, Germany) and mounted on microscope slides (ThermoFisher, Waltham, MA, USA). Deparaffinization was performed with xylene (Carl Roth, Karlsruhe, Germany), ethanol (Carl Roth), and distilled water. Following two PBS (Gibco, Carlsbad, CA, USA) washes, sections were covered with 1 µM fluorophore-labeled ligand TAMRA-NDP-α-MSH in HBSS (Gibco) containing 0.1% BSA (Miltenyi Biotech, Bergisch Gladbach, Germany) for 20 min. Sections were washed twice with PBS and subsequent blocking of unspecific binding and permeabilization was performed for two hours using 3% BSA and 0.2% Tween20 (Sigma, Darmstadt, Germany) diluted in PBS. We found that permeabilization with Triton X-100 prevents ligand-binding, while permeabilization with saponin hampers with MCT8 staining. Therefore, employing Tween20 for permeabilization is crucial for successful staining of human tissue using fluorophore-labeled ligands. Rabbit anti-MCT8 (1:300, Atlas Antibodies, Bromma, Sweden, #HPA003353) was diluted in PBS supplemented with 1% BSA and incubated with the samples overnight at 4 °C. After three washing steps with PBS, samples were incubated for two hours at room temperature with donkey anti-rabbit-488 (Jackson ImmunoResearch, West Grove, PA, USA, #711-545-152) diluted 1:250 in PBS supplemented with 1% BSA. Samples were washed twice with PBS and Sudan Black autofluorescence quenching was performed for 30 min with 0.1% Sudan Black (Sigma) in 70% ethanol. After three PBS washes, nuclei were counterstained for 10 min with 4,6-diamidino-2-phenylindole (DAPI, Sigma) diluted 1:7500 in PBS. After two final washing steps with PBS, microscope slides were covered with cover slips (ThermoFisher) in RotiMount mounting medium (Carl Roth) and analyzed on a Leica SP8 laser scanning confocal microscope (scan rate: 10 Hz, laser intensity: 2%) using the Leica LAS X software Version 3.7.6.

### 4.3. Cell Culture 

The human embryonic kidney 293 (HEK293) cell line was purchased from ATCC (Manassas, VA, USA), authenticated by single nucleotide polymorphism analysis, and regularly tested for mycoplasma contamination. Cells were cultivated in GlutaMAX-containing minimal essential medium (MEM, Gibco) supplemented with 5% fetal bovine serum (FBS, Gibco) and 1% non-essential amino acids (Gibco) at 37 °C and humidified air containing 5% CO_2_.

For BRET assays, HEK293 cells were seeded in 12-well plates (ThermoFisher) for transfection (40,000 cells/well) and later re-seeded in white opaque 96-well plates for measurement (22,000 cells/well). For cAMP measurements via GloSensor™ (Promega, Mannheim, Germany) or AlphaScreen^®^ (PerkinElmer, Waltham, MA, USA), and total and cell surface expression (HiBiT assay, Promega), 15,000 cells per well were seeded in poly-L-lysine-coated (Sigma) 96-well plates and incubated for 24 h before transfection. GloSensor™ and HiBiT assays were performed in white opaque, poly-L-lysine-coated 96-well plates (Corning, NY, USA, #3917); for other assays, translucent 96-well plates (Sarstedt, Nümbrecht, Germany) were used. For IP1 assays, 1.25 × 10^6^ cells were seeded in 3.5 cm dishes (ThermoFisher) and transfected 24 h later. For cell surface biotinylation assays, 2.0 × 10^5^ cells per well were seeded in 6-well plates (ThermoFisher) and incubated for 48 h before transfection. 

### 4.4. Plasmid Construction 

For signaling assays, the eukaryotic expression vector high copy (hc)pcDps was used containing the human MCT8 (NM_006517) or MC4R (NM_005912) sequences. The MC4R plasmid additionally contained an N-terminal HA-tag (YPYDVPDYA). For mock transfections, empty hcpcDps without an insert was used. For cell surface and total expression analysis, the HiBiT vector pBiT3.1-N cloning vector (Promega) was used to tag MC4R. For interaction studies, NanoLuciferase^®^/HaloTag^®^ fusion vectors pFC14A and pFC32K (Promega) and inserts were cloned for C-terminal tagging according to the manufacturer’s protocol. For cell surface biotinylation assays, hcpcDps containing SNAP-tagged MC4R or N-terminally HA-tagged MCT8 was used. Briefly, the N-terminal HA-tag of MC4R in hcpcDps was changed to an N-terminal SNAP-tag bearing the IgK signal peptide (ETDTLLLWVLLLWVPGSTGD), and the long isoform of human MCT8 with an N-terminal HA-tag was cloned into hcpcDps. All constructs were verified by Sanger sequencing (Microsynth, Göttingen, Germany).

### 4.5. Determination of Protein–Protein Interaction via NanoBRET™ 

For BRET-based determination of an interaction between MC4R and MCT8, the NanoBRET™ assay (Promega) was performed. MCT8 was fused C-terminally with HaloTag^®^ (HT), acting as an acceptor, while MC4R, the positive control TSHR, and the non-interacting control protein Rab6b were coupled C-terminally with NanoLuc^®^ (NL) as a donor (see “Plasmid Construction”). Cells were transfected 4–6 h after seeding using 3 µL FuGene (Promega) per well. Donor and acceptor DNA were transfected in a ratio of 1:8 (100 ng:800 ng) in addition to 100 ng pGEM-3Zf(+) (purchased from Promega) as a carrier. 20 h after transfection, cells were harvested with trypsin/EDTA (Gibco), centrifuged at 130× *g* for 5 min, and re-suspended in Opti-MEM without phenol red (Gibco) supplemented with 4% FBS. Cells were adjusted to 2 × 10^5^ cells/mL, divided into two pools, and either DMSO (Sigma) or HT-Ligand 618 were added in a 1:100 dilution. 20,000 cells per well of each pool were re-seeded in triplicates in white 96-well plates (Corning, #3917) and incubated for 4–6 h at 37 °C and 5% CO_2_. Victor Nivo plate reader (PerkinElmer) was used to inject 25 µL/well of NanoGlo^®^ Substrate (previously diluted 1:100 in Opti-MEM without phenol red) and donor and acceptor emission was measured at 460 nm and 618 nm, respectively. Dividing acceptor emission by donor emission resulted in the calculated BRET ratio. Background bleed-through correction was performed by subtracting the background ratio calculated from the respective DMSO control. Finally, the unit was converted to milliBRET units (mBU) by multiplying the corrected BRET ratio by 1000.

### 4.6. Determination of cAMP Accumulation via GloSensor™ and AlphaScreen^®^ Assays 

To measure cAMP accumulation via GloSensor™, cells were seeded in white 96-well plates (see Section 4.3) and transfected at ca. 70% confluence using Metafectene (Biontex, Munich, Germany). In each well, the culture medium was replaced with 120 ng of either MC4R plasmid and empty mock plasmid hcpcDps or MC4R plasmid and MCT8 plasmid in a 1:1 ratio in addition to 60 ng pGloSensor-F22 cAMP plasmid (GenBank accession number GU174434, purchased from Promega) using 0.6 µL Metafectene diluted in MEM without supplements. Cells were equilibrated 48 h after transfection with CO_2_-independent MEM (Gibco) containing 10% FBS and 2% GloSensor™ reagent (Promega) for two hours in the dark. α-MSH (Sigma) was diluted in PBS containing 0.1% BSA and 1.7 mM calcium chloride (Carl Roth) and added to the cells for final concentrations ranging from 10^−6^ to 10^−12^ M immediately before measurement of luciferase light emission on a plate reader (Mithras LB 940, Berthold Technologies, Bad Wildbad, Germany). GraphPad Prism 8.4.3 (GraphPad Software, LLC, La Jolla, CA, USA) was used for calculation of the AUC.

For cAMP accumulation measurement via AlphaScreen^®^, cells were transfected in MEM with 45 ng of either MC4R + mock or MC4R+MCT8 and stimulated with α-MSH in a concentration range from 10^−6^ M to 10^−12^ M. The detailed protocol has been previously described [63].

### 4.7. IP-One Assays 

For IP1 accumulation measurement (PerkinElmer), HEK293 cells were transfected in supplement-free medium 24 h after seeding with 15 µg of DNA (MC4R, MCT8, CB1R, or mock vector in appropriate ratios) using metafectene according to the manufacturer’s protocol. The next day, cells were re-seeded in triplicates at 40,000 cells per well in white 384-well culture plates (PerkinElmer). The medium was discarded 24 hours later by inverting the plate and cells were stimulated with the indicated concentrations of α-MSH (Sigma) in 1X Stimulation Buffer containing 20 mM Lithium chloride (Carl Roth) and 0.1% BSA or Stimulation Buffer alone. For IP-One assays in the presence of an MCT8 inhibitor, 10 µM SC (MedChem Express, South Brunswick, NJ, USA) was added to 1X Stimulation Buffer. Cells were incubated at 37 °C, 5% CO_2_ for two hours and acceptor IP1-d2 and donor anti-IP1-Terbium-Cryptate were added according to the manufacturer’s protocol, including three wells without donor as Cryptate blank. The plate was incubated at room temperature for 1 h while shaking at 300 rpm. Subsequent measurement was performed on a Victor Nivo plate reader (Excitation: 320 nm, emission: 620 nm and 665 nm). The HTRF^®^ ratio was calculated by dividing the value of the biological response (measured at 665 nm) by the value of the internal reference (measured at 620 nm), multiplying by 10,000, and subtracting the average Cryptate blank. IP1 accumulation was interpolated using a standard curve with non-linear regression and subsequently transformed for final IP1 concentrations using Y = 10^Y^, followed by normalization to MC4R + mock. To test for normal distribution of the measurement data, the Anderson–Darling, D’Agostino–Pearson, Shapiro–Wilk, and Kolmogorov–Smirnov tests were performed using GraphPad Prism 8.4.3. All four tests validated the Gaussian distribution of the measured values. To visually test for normal distribution, a quantile–quantile plot (Q–Q plot) was graphed, comparing theoretical residuals with actual sample residuals. The data points on the Q–Q plot aligned with the identity line, therefore supporting the previously performed mathematical analyses of normal distribution. Based on these analyses, a one-way ANOVA was performed for comparison of α-MSH-induced IP1 formation in MC4R + mock-transfected cells and MC4R + MCT8/CB1R-transfected cells.

### 4.8. Cell Surface Biotinylation Assays and Western Blot Analysis 

HEK293 cells were transfected with 1 µg of each plasmid, either SNAP-MC4R + mock, HA-MCT8 + mock, or SNAP-MC4R + HA-MCT8 using Metafectene in accordance with the manufacturer’s protocol. Two wells of a 6-well plate were transfected for each condition. The transfection medium was replaced with complete MEM after 24 h. At 48 h post-transfection, cells were rinsed once with ice-cold PBS and then incubated with 1 mM EZ-Link™ Sulfo-NHS-SS-Biotin (ThermoFisher) in PBS at 4 °C for 30 min. The biotinylation reagent was quenched with a 50 mM Tris (pH 8.0) buffer. Cells (two wells per condition) were collected by scraping and pelleted at 6000× *g*, 4 °C for 5 min. The pellets were washed once with PBS and subjected to streptavidin pulldown.

Cells were lysed in 100 µL of PBS containing 1% Triton X-100 (Sigma) and protease inhibitor (ThermoFisher). The lysates were clarified by centrifugation at 7000× *g*, 4 °C for 15 min, and the protein concentrations were determined using the BCA assay (ThermoFisher). Then, 6 µg of each lysate was stored as a total lysate sample, and the remaining lysates were incubated with equilibrated Streptavidin Plus UltraLink™ beads (ThermoFisher) overnight at 4 °C with gentle rotation. Unbound samples were removed by centrifugation at 7000× *g* for 2 min. The beads were washed three times with PBS containing 0.5% SDS and protease inhibitor (ThermoFischer). Biotinylated cell surface proteins were eluted from the beads with 2X Laemmli buffer containing 100 mM DTT at 70 °C for 20 min with gentle agitation.

For Western blot analysis, the total lysate and cell surface proteins were separated on an 8% SDS–PAGE gel and transferred onto a nitrocellulose membrane (BioRad, Hercules, CA, USA). After a 1-h incubation with BSA blocking solution, the membranes were incubated with primary antibodies overnight at 4 °C and with secondary antibodies at room temperature for 1.5 h. TBST buffer was used to rinse the membranes after antibody incubations. Proteins were visualized using Lumi-Light Western blotting substrate (Roche) on a ChemiDoc imaging system (BioRad). Anti-SNAP (New England BioLabs, P9310), anti-HA (Roche, 12CA5), anti-pan-Cadherin (Cell Signaling Technologies, Danvers, MA, USA, #4068), HRP-conjugated anti-mouse (Agilent Technologies, Santa Clara, CA, USA, P0447) and anti-rabbit (Agilent Technologies, P0448) antibodies were used at a 1:1000 dilution.

### 4.9. Analysis of Cell Surface and Total Expression via HiBiT Assay 

For cell surface and total expression of MC4R, split-luciferase-based assays using the NanoGlo^®^ HiBiT detection system (Promega) were performed in HEK293 cells expressing low amounts of the HiBiT-tagged receptor in the absence or presence of MCT8. This split luciferase-based assay employs a short peptide tag (HiBiT), which emits luminescence when interacting with its complement (LargeBiT) and can be used for the quantification of a HiBiT-tagged protein in whole cell lysates as well as on the cell surface. Measurement was performed according to the manufacturer’s protocol (rapid measurements protocol). The detailed protocol has been previously described [33].

## 5. Conclusions

Heterodimerization of GPCRs can have significant influences on their trafficking to the cell surface, ligand binding, G-protein coupling, and internalization. In this study, we demonstrated that the thyroid hormone transporter MCT8 interacts with the neural MC4R in the human hypothalamus and modulates MC4R-induced PLC activation. We showed this interaction not only by using in vitro assays but also by developing a novel fluorophore-labeled ligand staining procedure, demonstrating that the receptor and transporter also co-localize in a physiological setting in neurons of the human PVN. With MC4R as a promising target for anti-obesity treatments, this investigation of MC4R interaction partners can be of relevance for the development of clinical therapies. The mechanism by which MCT8 influences PLC activation of the MC4R and its functional relevance in physiology remains subject to further investigation, yet this study demonstrates the importance of understanding GPCR heterodimerization to obtain a comprehensive understanding of GPCR action in health and disease.

## Data Availability

Data are contained within the article or Supplementary Materials. A source data file is available for download.

## Supplementary Materials

The following supporting information can be downloaded at: https://www.mdpi.com/article/10.3390/ijms25147565/s1.
