# Supplementary material for: Melanocortin-4 Receptor PLC Activation Is Modulated by an Interaction with the Monocarboxylate Transporter 8
Source: Int J Mol Sci. 2024 Jul 10;25(14):7565. doi: 10.3390/ijms25147565 (PMC11277258; doi:10.3390/ijms25147565)
Supplement: Supplementary file 1 [file ijms-25-07565-s001.zip › ijms-3082857-supplementary.pdf]

Supplementary Materials

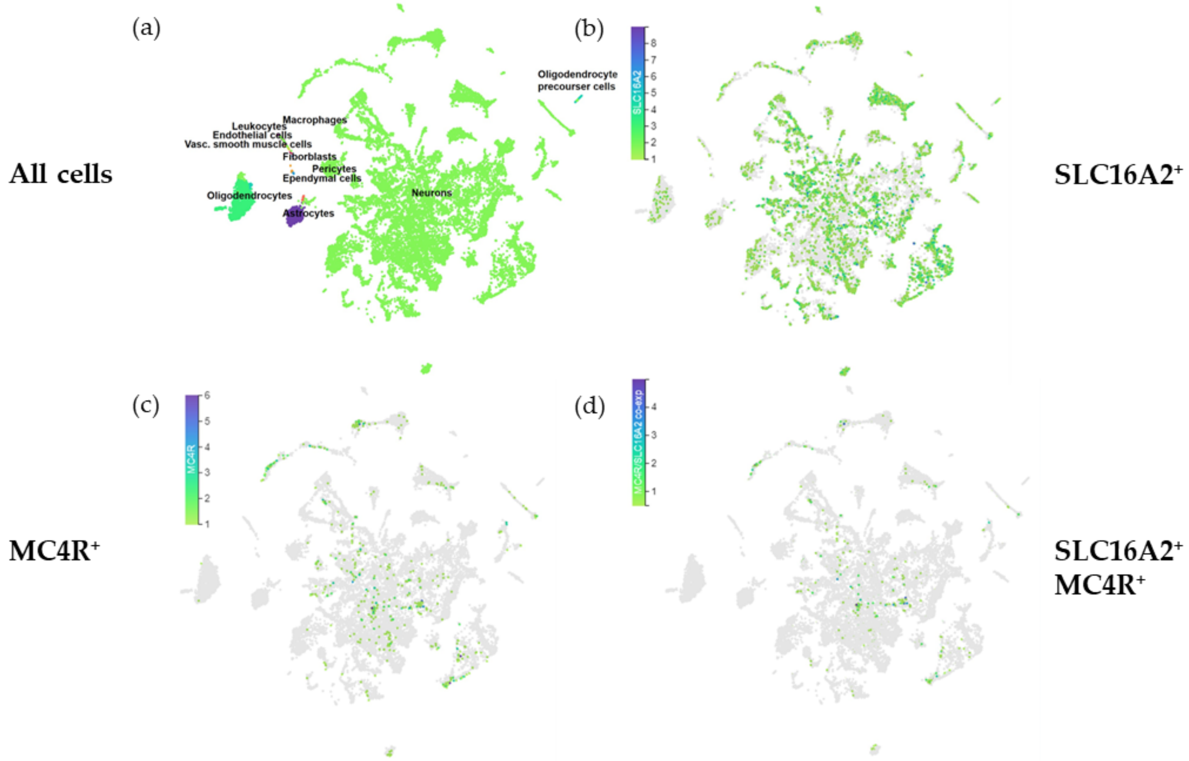

Figure S1. Analysis of *MC4R* and *SLC16A2* expression in a publicly available single-cell RNA-sequencing data set of the human hypothalamus. We found co-expression of *MC4R* and *SLC16A2,* the MCT8-encoding gene, in neurons of dissected human hypothalamus (dataset from [30]). Several nuclei were sequenced, including the paraventricular nucleus. While most sequenced cells had neuronal identity **(a)**, sorting for *SLC16A2*-expressing cells (mean expression cut-off set to 1) revealed *SLC16A2* expression in various cell types, including neurons, epithelial cells, and oligodendrocytes **(b)**. Additional sorting for *MC4R*-expressing cells, which were of neuronal identity only, showed expression in fewer amounts of cells **(c)**. Within this sub-population of neurons, we sorted for *SLC16A2* expression and found that around 40% of the *MC4R*-expressing cells co-express *SLC16A2* **(d)**.

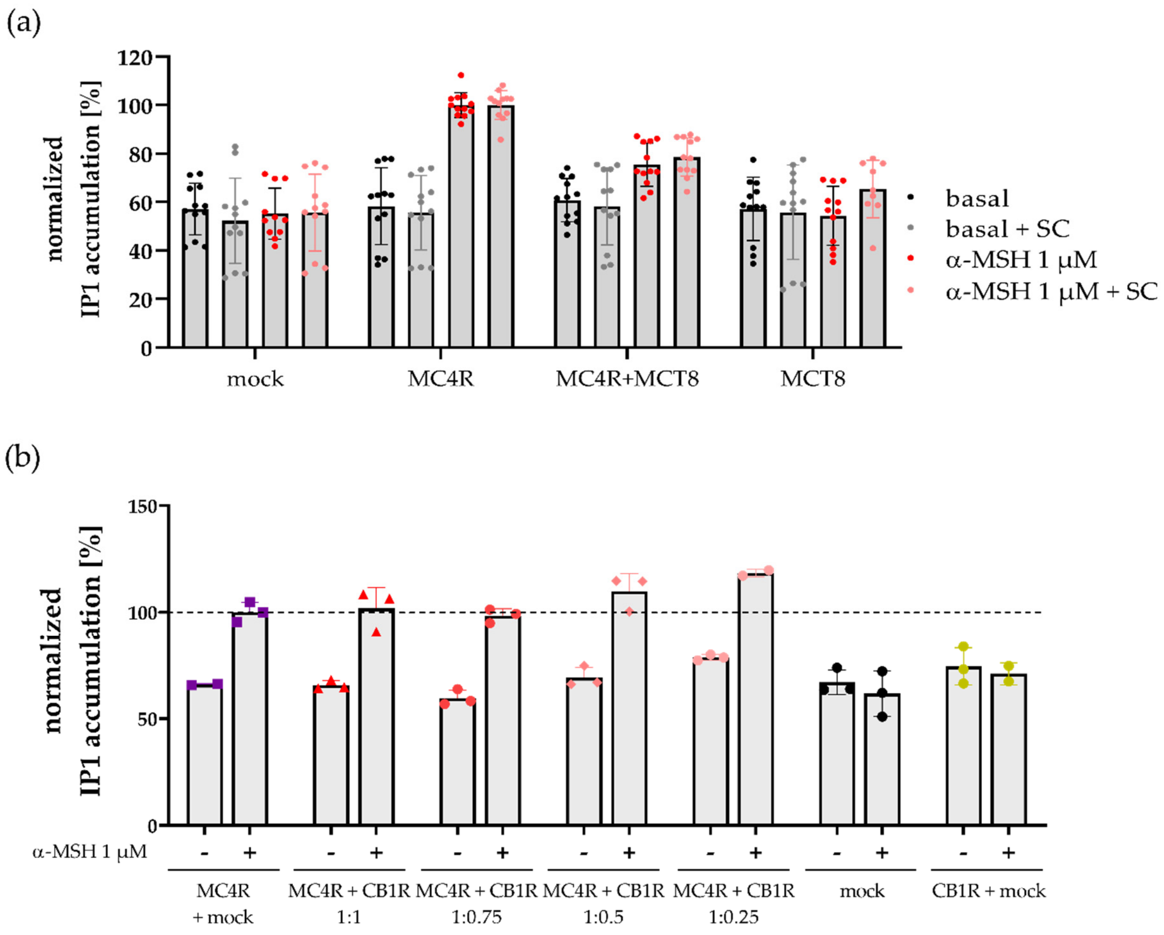

Figure S2. IP-One assay with silychristin (SC) and IP-One control assay with MC4R and CB1R. (a) HEK293 cells were co-transfected with MC4R + mock or MC4R + MCT8 in a 1:1 ratio and IP1 accumulation was measured in the presence and absence of the MCT8-specific inhibitor SC (10 μM) in basal conditions and after stimulation with 1 μM a-MSH. No significant differences were detected between the presence of SC in comparison to the absence of SC, neither in basal nor in stimulated conditions. Values represent individual measurement values ± SD from four independent experiments with three technical replicates. Statistical analysis was performed using a two-way ANOVA with Tukey's multiple comparison test with statistical significance set to * $p < 0.05$. **(b)** HEK293 cells were co-transfected with MC4R + mock or MC4R + CB1R in different ratios (indicated on the x-axis), and IP1 accumulation was measured after stimulation with 1 μM α-MSH to investigate if the effects seen for IP1 accumulation in MC4R-MCT8 transfected cells are specific to MCT8. No differences in IP1 accumulation were detected for either condition, nor for mock- and MCT8 + mock transfected samples. IP1 accumulation was also measured in basal conditions with no differences in all transfections. Values represent individual measurement values from one assay with three technical replicates.

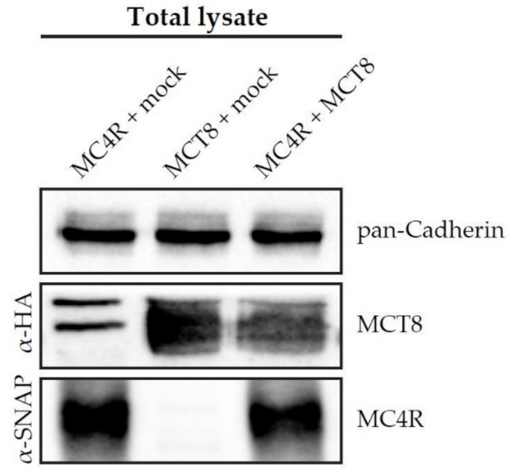

Figure S3. Western blot analysis of total MC4R and MCT8 expression in cell lysates of co-transfected HEK293 cells. HEK293 cells were transfected with SNAP-tagged MC4R + mock, HA-tagged MCT8 + mock or co-transfected with SNAP-MC4R and HA-MCT8. Western blotting of whole cell lysates demonstrated no obvious differences in total expression of MC4R or MCT8 in the presence or absence of one another. Due to nonspecific binding of the anti-HA antibody in MC4R + mock sample, quantification was not performed. However, these nonspecific bands were not detectable in the Western blot analysis of cell surface samples, implying the successful isolation of biotinylated cell surface proteins from cytosolic contaminants.
